# Tumor-associated autoantibodies in combination with alpha-fetoprotein for detection of early stage hepatocellular carcinoma

**DOI:** 10.1371/journal.pone.0232247

**Published:** 2020-05-06

**Authors:** Christopher Welberry, Isabel Macdonald, Jane McElveen, Celine Parsy-Kowalska, Jared Allen, Graham Healey, William Irving, Andrea Murray, Caroline Chapman

**Affiliations:** 1 Oncimmune ltd, Nottingham, United Kingdom; 2 School of Medicine, University of Nottingham, Nottingham, United Kingdom; 3 NIHR Nottingham Biomedical Research Centre, Nottingham University Hospitals NHS Trust and the University of Nottingham, Nottingham, United Kingdom; 4 Bowel Cancer Screening Program, Nottingham University NHS Trust, Nottingham, United Kingdom; University of Cambridge, UNITED KINGDOM

## Abstract

**Background:**

Hepatocellular carcinoma (HCC) continues to be a leading challenge in modern oncology. Early detection via blood-based screening tests has the potential to cause a stage-shift at diagnosis and improve clinical outcomes. Tumor associated autoantibodies (TA-AAbs) have previously shown the ability to distinguish HCC from patients with high-risk liver disease. This research aimed to further show the utility of TA-AAbs as biomarkers of HCC and assess their use in combination with Alpha-fetoprotein (AFP) for detection of HCC across multiple tumor stages.

**Methods:**

Levels of circulating G class antibodies to 44 recombinant tumor associated antigens and circulating AFP were measured in the serum of patients with HCC, non-cancerous chronic liver disease (NCCLD) and healthy controls via enzyme-linked immunosorbent assay (ELISA). TA-AAb cut-offs were set at the highest Youden’s J statistic at a specificity ≥95.00%. Panels of TA-AAbs were formed using net reclassification improvement. AFP was assessed at a cut-off of 200 ng/ml.

**Results:**

Sensitivities ranged from 1.01% to 12.24% at specificities of 95.96% to 100.00% for single TA-AAbs. An ELISA test measuring a panel of 10 of these TA-AAbs achieved a combined sensitivity of 36.73% at a specificity of 89.89% when distinguishing HCC from NCCLD controls. At a cut-off of 200 ng/ml, AFP achieved a sensitivity of 31.63% at a specificity of 100.00% in the same cohort. Combination of the TA-AAb panel with AFP significantly increased the sensitivity for stage one (40.00%) and two (55.00%) HCC over the TA-AAb panel or AFP alone.

**Conclusions:**

A panel of TA-AAbs in combination with AFP could be clinically relevant as a replacement for measuring levels of AFP alone in surveillance and diagnosis strategies. The increased early stage sensitivity could lead to a stage shift with positive prognostic outcomes.

## Introduction

Hepatocellular carcinoma (HCC) is one of the major challenges of modern oncology. It is the sixth most common cancer worldwide and the fourth most common cause of cancer related mortality [1]. The majority of cases occur in countries with high prevalence of viral hepatitis, such as China, Japan and Egypt. However; rates in western countries are on the rise, attributed to modern lifestyle changes such as increased alcohol consumption and poor diets.

Current American Association for the Study of Liver Disease (AASLD) recommendations on surveillance of HCC are for ultrasound (US) imaging, with or without serum Alpha-fetoprotein (AFP) measurement for only the highest risk patients [2]. The largest randomised control trial (RCT) for surveillance of HCC, using AFP and US, analysed 18,816 patients with HBV infection or a history of chronic hepatitis from China and showed a clear benefit of reduction in stage at diagnosis and mortality rates for the patients undergoing surveillance [3]. Presentation at earlier stage is linked with increased overall survival, however this is not the only outcome required to implement screening strategies. A systematic review of HCC screening studies concluded that HCC screening by US is possible at a reasonable cost per quality adjusted life year gained (QALY), but the authors highlight the need for an appropriate randomised controlled trial (RCT) to confirm the results [4].

Whilst US based surveillance strategies have shown increased overall survival rates at an acceptable cost per QALY, this type of imaging still suffers from a poor ability to detect early stage HCC. A meta-analysis found the pooled sensitivity of US for early stage HCC to be 45% at a high specificity of 92% with addition of AFP showing significant increase in early stage sensitivity to 63% but with a trade-off in reduced specificity to 84% [5]. AFP testing alone also struggles to detect early stage disease, with sensitivity typically below 50% [6,7] at specificities of 80–94% in non-cancerous chronic liver disease (NCCLD) control groups [8].

Autoantibody (AAb) production to tumor associated antigens (TAAs) has been extensively described in cancer patients [9] and is thought to be triggered by the proinflammatory nature of tumor establishment and growth [10]. These tumor associated autoantibodies (TA-AAbs) are primarily targeted at three forms of TAAs [10]; mutated proteins, aberrantly expressed proteins and post-translationally modified proteins [11]. However, the exact role of AAbs, and the immune system as a whole, in tumor control and progression is still debated [10].

The TAAs which produce an AAb response in HCC, and other malignancies, are wide ranging and there are numerous studies that report the existence of different TAAs that can elicit an AAb response [12–21]. In heterogeneous diseases, such as cancer, each subtype may have its own set of unique biomarkers where the sensitivity of a single marker for the disease is capped by the prevalence of that subtype [22–25]. It has also been shown that a panel of multiple TAAs, rather than a solitary marker, can be useful in detecting HCC [15,26,27].

This study aims to determine whether measuring autoantibodies against novel TAAs can distinguish HCC patients from those with NCCLD and healthy controls. Assessment of whether their measurement in combination with AFP may enhance diagnosis of HCC in multiple disease stages, as previously demonstrated [28] will also be performed.

## Materials and methods

### Antigen selection

Antigens for the Discovery study were selected from two searches of Embase, Web of Science and Medline: (i) a search for TAAs with existing TA-AAb data in HCC patients, and (ii) a search for review papers of proteins that are highly associated with HCC (exact search terms are shown in S1 Table). From these two searches, potential antigens that may elicit an AAb response were identified based on the strength of their association with HCC or previously demonstrated immunogenicity in HCC patients compared to controls. Antigens/TA-AAbs which overlapped with our previous study [15] were removed from the review so as to increase the number of new markers tested in this Discovery study. For the Confirmation study, six additional antigens with high Youden’s J statistic were selected from the previous study [15] to analyse the potential combination with leads from this Discovery study.

### Protein production

The cDNA for the TAAs, were sub-cloned into modified pET21b or pET45b vectors to create TAA-BirA-6xHis fusions, as previously described [29]. TAA fusion vectors plus a fusion tag only control were transformed into BL21(DE3) *Escherichia Coli* competent cells [29] and cultured as either 60 ml (Discovery study) or 500 ml (Confirmation study) volumes of TB overnight express auto-induction medium (Novagen). For NY-ESO-1, EpCAM, VIM and Annexin II, 500 ml cultures were used for the Discovery study instead of 60 ml. Proteins were purified as previously described using either Ni Sepharose 6 Fast Flow resin (GE Healthcare) in 24 well filter plates [29] (Discovery study) or 5 ml HisTrap FF crude prepacked columns (GE healthcare) [30] (Confirmation study). Proteins were analyzed for molecular weight and purity using SDS-PAGE and Western Blotting and quantified by Bradford assay (Biorad).

### ELISA

Autoantibodies were detected by ELISA according to previously described methods [30]. Briefly, TAAs plus a fusion tag only (BirA-6xHis) control were coated on to the wells of microtiter plates at either two (160 nM and 50 nM, Discovery study) or five concentrations (160 nM, 50 nM, 16 nM, 5 nM and 1.6 nM, Confirmation study) in duplicate. All patient specimens were diluted 1 in 110 in blocking buffer and allowed to react with the immobilised TAAs. After plate washing, the presence of IgG AAbs was detected using horseradish peroxidase-conjugated rabbit anti-human IgG (Dako), 3,3′,5,5′-tetramethylbenzidine chromogenic substrate (Merck) and absorbance/optical density (OD) measured at 650 nm wavelength. Circulating AFP was measured using a commercially available ELISA (Aviva Systems Biology, OKBA00002).

### Cohort

All patient specimens were collected with written informed consent at the respective collection centres. Approval from the University of Nottingham Medical Research Ethics Committee was also obtained. Ethical approval was obtained from the Trent Research Ethics Committee (10/H0405/41) and the University of Nottingham Medical School Research Ethics Committee (BT/07/2007). Approval was also obtained from Nottingham University Hospitals through the Comprehensive Local Research Network (CLRN) identification 43961.

Serum or plasma samples from three patient groups were used in this study: (i) patients diagnosed with HCC (purchased from Indivumed GmbH, n = 100); (ii) control cohort of patients with NCCLD, consisting of liver cirrhosis, alcoholic liver disease and chronic viral hepatitis (collected by the Queens Medical Centre Biomedical Research Unit, Nottingham, n = 115); (iii) control cohort of healthy individuals (collected as described previously [31], n = 99). All HCC cohort patients were matched by both age and sex to the healthy control cohort, however only gender matching to the NCCLD cohort was possible. The cohorts were used for both the Discovery and Confirmation studies with the following modifications (i) Sixteen samples in the NCCLD control cohort for the Discovery study (n = 99) were replaced for the Confirmation study due to volume restrictions (ii) One sample in the HCC cohort for the Discovery study (n = 99) was replaced and one removed for the Confirmation study due to volume restrictions. Demographics of all samples plus tumor/node/metastasis (TNM) stage [32] of HCC samples are shown in Table 1. The HCC samples were obtained from a commercial supplier with limited metadata provided, tumor stage was provided using the TNM system rather than BCLC and no data regarding overall liver status, such as Child-Pugh scores, were available. Aetiology of the NCCLD controls is shown in S2 Table.

**Table 1:** Demographics of the cohorts utilised in the Discovery and Confirmation ELISA.

	Discovery	Confirmation^a^
HCC		
Number	99	98
Median age	63	63
Age range	30–91	30–91
% Male	69	69
TNM stage		
1	35	35
2	21	20
3	21	21
4	2	2
N/A	20	20
Primary tumor size (cm)		
Samples with data available	88	87
Mean	5.9	6.02
Min	0.4	0.4
Max	19	19
NCCLD		
Number	99	99
Median age	57	50
Age range	30–89	30–82
% Male	69	69
Healthy		
Number	99	99
Median age	63	63
Age range	30–87	30–84
% Male	69	69

^a^The Confirmation cohort is primarily the same as the Discovery apart from the changes described in the text. N/A = TNM stage data unavailable.

### Data analysis

For the AAb ELISA, signal for the fusion tag only control protein was subtracted from the raw OD value of each TA-AAb to correct for non-specific binding to bacterial contaminants. In the Confirmation study ELISA, samples were excluded based on a visual assessment of the duplicate titration curve data, if anomalous results were observed based on the surrounding points of the curve which could not be resolved by the exclusion of a single replicate, or if high binding was observed in the fusion tag only control wells. Cut-offs were optimised for differentiating HCC from NCCLD controls. The sensitivity, specificity and Youden’s J statistic [33] for HCC vs NCCLD controls were calculated for each TA-AAb at each sample OD. An algorithm assigned cut-offs to each TA-AAb at 160 nM and 50 nM data points under the following criteria: the OD value which gave the highest Youden’s J statistic ≥0.00 with a specificity ≥95%. Panels of TA-AAbs were optimised using net reclassification improvement (NRI) [34] using a custom Stata script (Stata, StataCorp LLC). Starting with the TA-AAb with the highest Youden’s J statistic, TA-AAbs were added one at a time based on the highest NRI score of those remaining, after each addition the NRI scores were recalculated until no further improvement to the score of the panel could be achieved. For the AFP ELISA, samples above 200 ng/ml were deemed positive for AFP and statistical analysis was performed in Stata. Significance of the differentiation of HCC from NCCLD controls was assessed using the methods described by Delong et al [35]. Significance of panel association with stage was analysed using logistic regression and significance of sensitivity difference for individual HCC stages between tests was analysed using McNemar’s test [36]. Specificity differences between groups was analysed using Pearson Chi-squared analysis. Dot plots to show distribution of ODs by group were constructed in GraphPad Prism 6 (GraphPad Software). K-fold cross-validation was performed in Stata for a choice of k = 5 [37].

## Results

### Antigen selection and production

After removal of antigens which overlapped with our previous study [15], the literature review returned 64 unique antigens from 20 papers which have potential to generate TA-AAbs during HCC tumorigenesis. After removing antigens not suitable for bacterial expression (carbohydrate antigens), 20 proteins from search (i) and 25 additional proteins from search (ii) were selected. Of these 45 proteins, one (HGF) failed cloning and was therefore not included in these studies, leaving 44 antigens to be used in the Discovery study ELISA (S3 Table). All proteins were successfully expressed and purified which was confirmed by detection at the correct molecular weight by Coomassie stained SDS-PAGE gels and/or Western blot.

### Discovery study

**Fig 1:**
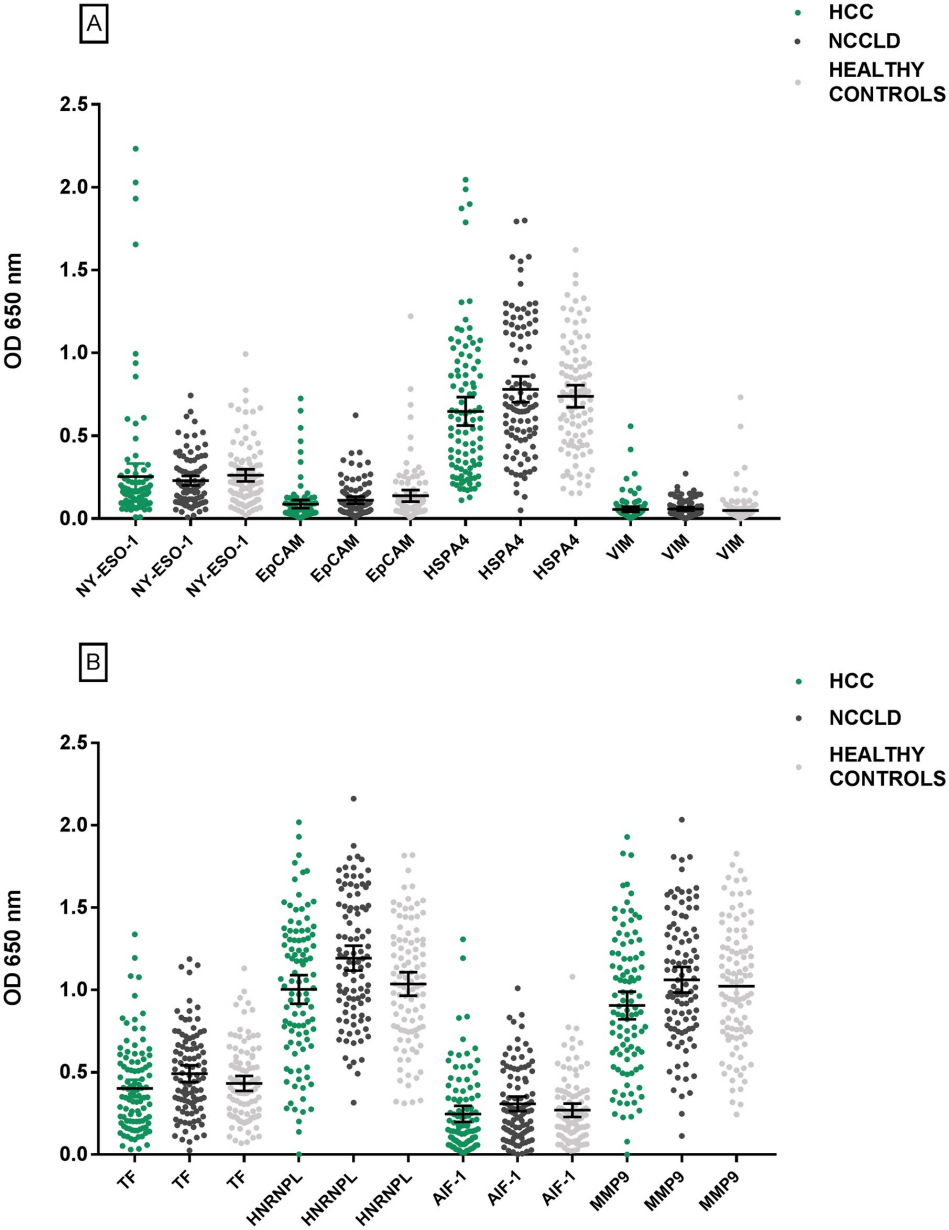
Dot plots of OD signals for each TA-AAb in the Discovery cohort panel. Error bars signify the mean with 95% confidence intervals.

For the ELISA of the Discovery cohort, cut-offs were assigned for all TA-AAbs for at least one concentration, except CK19 which was excluded due to the fact that no appropriate cut-offs could be identified. Sensitivities ranged from 1.00% to 7.10%, specificities from 96.00% to 100.00% for the NCCLD samples and Youden’s J statistic from 0.00 to 0.07 (S3 Table). TA-AAbs to NY-ESO-1 were the most discriminatory for differentiating HCC patients from NCCLD patients (Youden’s J statistic 0.07, see Fig 1). NRI analysis with a Youden’s J statistic ≥0.00 established a panel of eight TA-AAbs (Table 2). Dot plots representing the OD value for each TA-AAb in the panel are shown in Fig 1. Sensitivity for HCC of this panel was 28.28%, with specificities in the NCCLD and healthy control groups of 93.94% and 87.88% respectively. Ability to distinguish HCC from the NCCLD control group was significantly greater for the panel over the most discriminatory single marker, NY-ESO-1 coated at 160 nM (p = 0.0015).

**Table 2:** NRI Discovery study TA-AAb panel.

Addition order	Plate coating antigen	Antigen concentration (nM)	Sensitivity (%)	Specificity NCCLD (%)	Specificity Healthy (%)
1	NY-ESO-1	160	7.07	100.00	98.99
2	EPCAM	50	5.05	98.99	93.94
3	HSPA4	50	4.04	100.00	100.00
4	VIM	160	7.07	97.98	93.94
5	HNRNPL	160	3.03	98.99	100.00
6	TF	160	2.02	100.00	100.00
7	AIF-1	50	2.02	100.00	100.00
8	MMP9	160	3.03	98.99	98.99
Panel of 1–8			28.28	93.94	87.88

Optimally performing TA-AAb ELISA panel determined by NRI analysis for the Discovery study.

### Confirmation study

The Discovery panel of eight TA-AAbs along with an additional seven leads from the Discovery ELISA which were thought to be gender specific and six leads identified from previous work [15] (21 in total, S4 Table) were assessed by ELISA for the Confirmation cohort. After quality checks of the ELISA data, four of the healthy cohort control samples were excluded from the analysis due to non-specific binding. TA-AAbs cut-offs were assigned for 15 of the 21 for at least one antigen plate coating concentration, six of the 21 (TA-AAbs to CDKN1B, GBU4-5, NPM1, VIM, HSPD1 and HNRPL) had no ability to distinguish HCC patients from controls for this cohort.

**Fig 2:**
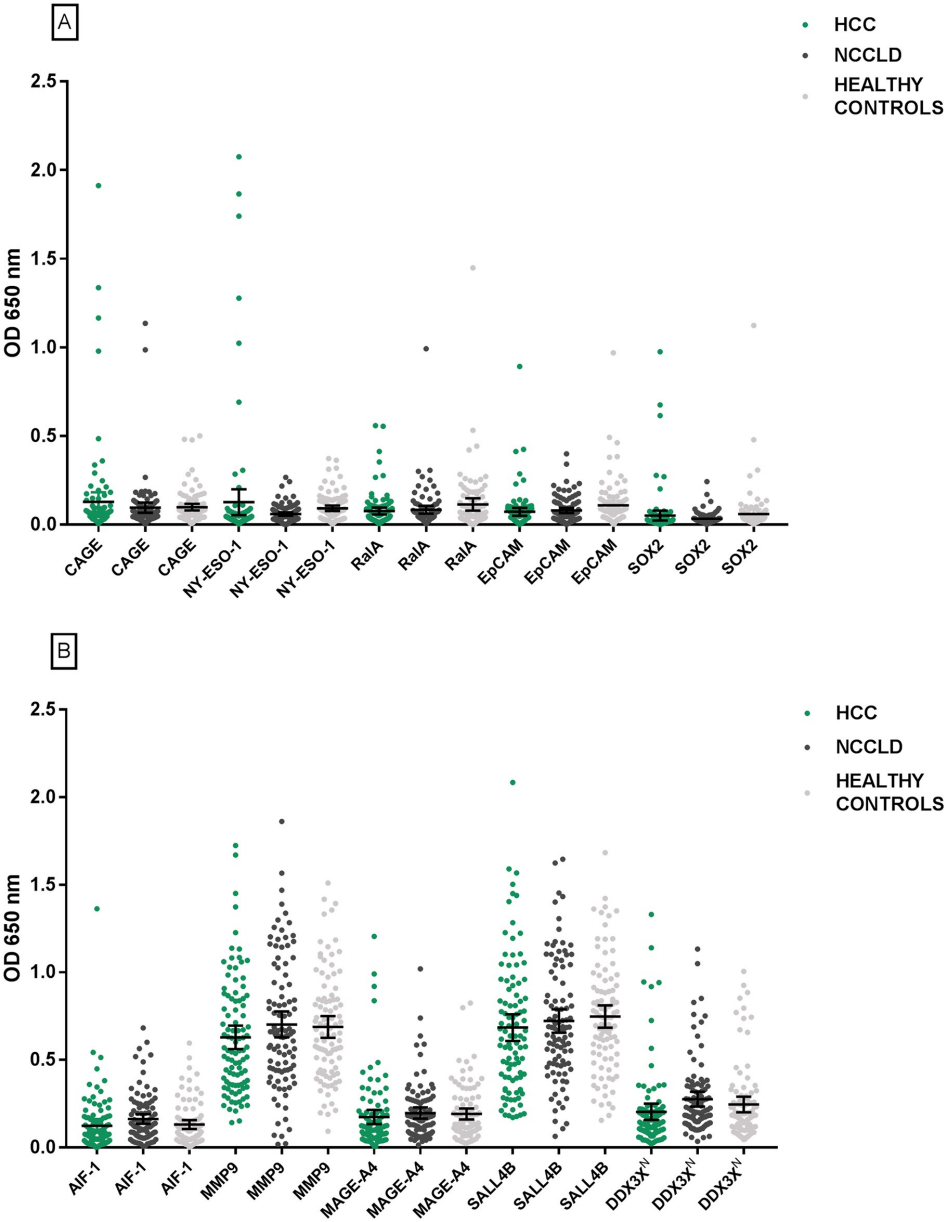
Dot plots of OD signals for each TA-AAb in the Confirmation cohort panel. Error bars signify the mean with 95% confidence intervals. ^N^ indicates the N-terminal tagged version of DDX3X, see S3 Table for more information.

HCC sensitivities ranged from 1.02% to 12.24% for individual TA-AAbs, specificities ranged from 95.96% to 100.00% for the NCCLD cohort and Youden’s J statistic from 0.00 to 0.09 (S4 Table). TA-AAbs binding to CAGE were the most discriminatory for distinguishing HCC patients from those with NCCLD (Youden’s J statistic 0.09). NRI analysis of the data with a Youden’s J statistic ≥0.00 established a panel of 10 TA-AAbs (Table 3). This panel had sensitivity for HCC of 36.73% with specificities in the NCCLD and healthy control groups of 89.89% and 84.21% respectively. At a prevalence of 2.40% [38], the positive predictive value (PPV) and negative predictive value (NPV) of the TA-AAb panel were 8.21% and 98.30% respectively for HCC vs NCCLD. Area under the curve (AUC) was calculated as 0.616 for differentiating HCC from NCLLD and five-fold cross validation of the AUC was 0.629 ± 0.062. Specificity of the 10 TA-AAb panel was not significantly different between NCCLD patients with hepatis B compared to hepatitis C (p = 0.72). Dot plots representing the OD value for TA-AAbs to each antigen in the panel are shown in Fig 2. Ability to distinguish HCC from NCCLD controls was significantly greater for the 10 TA-AAb panel over the most discriminatory single marker, CAGE coated at 50 nM (p = 0.0002).

**Table 3:** NRI Confirmation study TA-AAb panel.

Addition order	Plate coating antigen	Antigen concentration(nM)	Sensitivity (%)	Specificity NCCLD (%)	Specificity healthy controls (%)
1	CAGE	50	12.24	96.97	90.53
2	NY-ESO-1	50	8.16	100.00	95.79
3	RalA	50	4.08	98.99	95.79
4	EpCAM	50	3.06	100.00	96.84
5	SOX2	160	3.06	100.00	98.95
6	AIF-1	160	1.02	100.00	100.00
7	MMP9	160	2.04	98.99	100.00
8	MAGE-A4	50	4.08	98.99	100.00
9	SALL4B	160	6.12	96.97	98.95
10	DDX3X^N^	160	5.10	97.98	97.89
Panel of 1–10			36.73	89.90	84.21

Optimally performing TA-AAb ELISA panel determined by NRI analysis for the Confirmation study.

^N^ indicates the N-terminal tagged version of DDX3X, see S3 Table for more information.

### AFP ELISA

To compare the performance of circulating AFP and its potential combination with the 10 TA-AAb panel, levels were measured for the Confirmation cohort. Using a cut-off of 200 ng/ml, the AFP ELISA had a sensitivity of 31.63% for HCC with specificities of 100.00% in both the NCCLD and healthy control groups. Addition of AFP to the Confirmation study panel of TA-AAbs for the Confirmation cohort raised sensitivity from 36.73% to 55.10% with no change in specificity for either control group. At a prevalence of 2.40% [38], the PPV and NPV of the TA-AAb panel plus AFP were 11.83% and 98.79% respectively. The Confirmation study TA-AAb panel plus AFP was significantly better at distinguishing HCC from NCCLD samples than the TA-AAb panel (p<0.0001) or AFP (p = 0.0122) alone. When separated by AFP positivity, the TA-AAb panel had an overall sensitivity of 41.94% in AFP positive samples and 34.33% in AFP negative samples. TA-AAbs for CAGE remained the highest sensitivity at 11.94% and AIF-1 was the only TA-AAb to be redundant in the AFP negative group (S4 Table).

### HCC stage analysis

**Fig 3:**
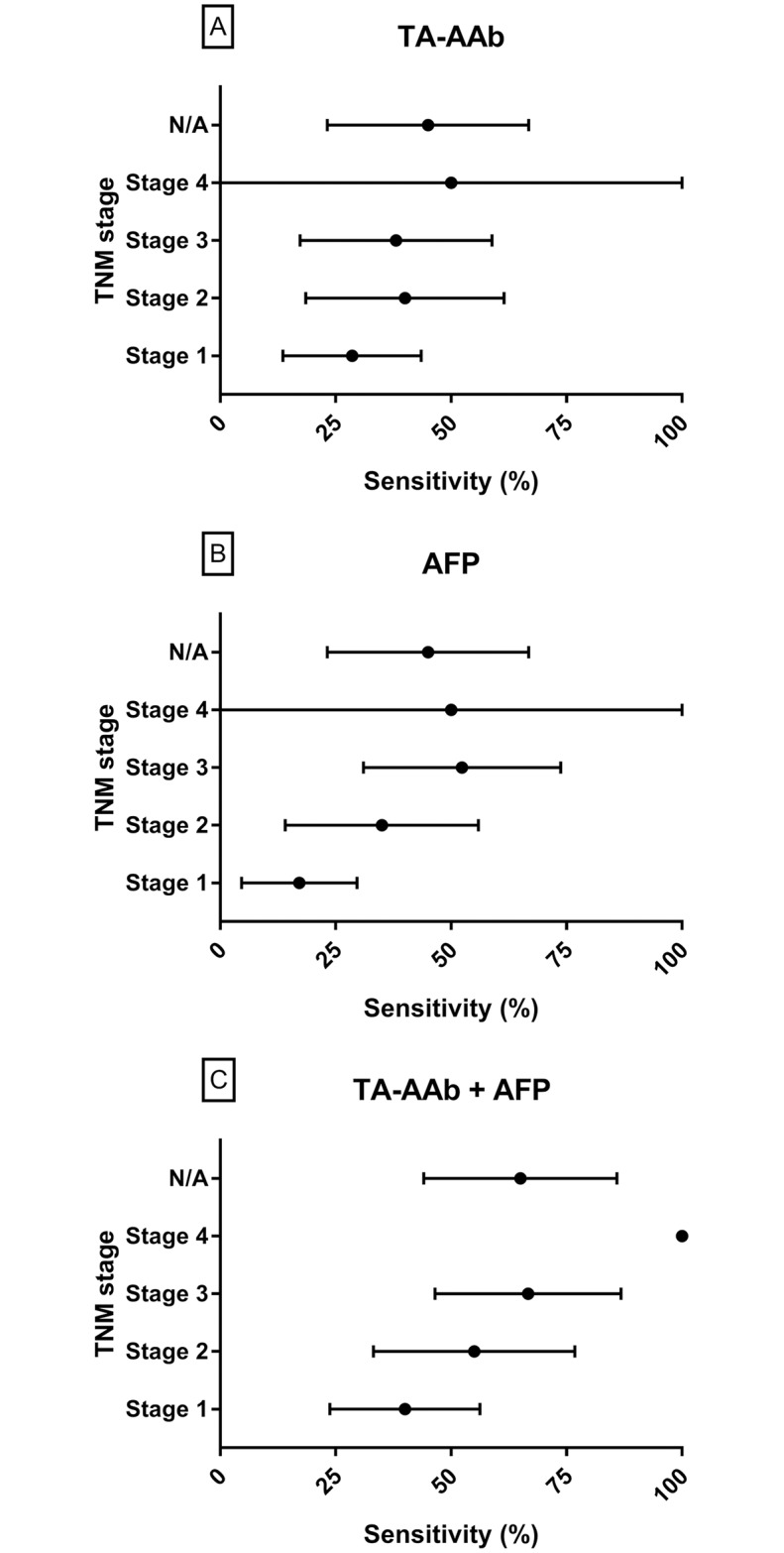
Forest plots of the sensitivity of the Confirmation TA-AAb panel (A), AFP (B) and a combination of both (C) by HCC TNM stage. N/A indicates sensitivity for samples of an unknown HCC stage.

The sensitivity of the TA-AAb Confirmation study panel, AFP and a combination of both by stage are shown in Fig 3. TA-AAb panel sensitivity was not significantly associated with increasing stage (p = 0.36) whereas AFP sensitivity was significantly associated with increasing stage (p = 0.016). The combination of the TA-AAb panel and AFP was significantly associated with increasing stage (p = 0.022). Although addition of AFP to the TA-AAb panel led to a significant association of sensitivity with increasing stage, sensitivity for stage 1 and 2 (early stage) HCC was significantly higher, at 45.45%, for the combined test than either the TA-AAb panel, at 32.72%, (p = 0.0082) or AFP, at 23.63%, (p = 0.0005) alone. Sensitivity of the TA-AAb panel alone for early stage samples in AFP negative patients was 27.59%. vs 46.15% for AFP positive patients.

## Discussion

The findings that single TA-AAb sensitivities for distinguishing HCC from high risk control cohorts are low is not surprising and has been reported previously by ourselves and others [15,20,39,40]. As with many cancers, HCC displays a high degree of molecular heterogeneity attributed to the complex interactions of various underlying liver diseases [41] and therefore a panel of biomarkers which reflects this heterogeneity may be of more use than one biomarker alone. It is quickly becoming the consensus that biomarker test development for many cancers will require the use of a panel of biomarkers from multiple molecular pathways. A TA-AAb signature has previously been shown to be additive to circulating PSA levels to differentiate prostate cancer from benign prostatic hyperplasia [42] and a combination of three circulating tumor proteins with one TA-AAb has been shown to be useful for the early detection of non-small cell lung cancer [43].

Fifteen antigenic leads from the Discovery study described here plus six identified in our previous studies [15], twenty one in total, were produced on a larger scale and retested on predominantly the same cohort (Confirmation) to verify leads on an independent protein batch. After this process, five leads identified during the Discovery study (CDKN1B, NPM1, VIM, HSPD1 and HNRNPL) and one lead from our previous work (GBU4-5) no longer showed ability to distinguish HCC from NCCLD samples. Combining individual TA-AAbs from the remaining 15 into a panel, using NRI, established a panel of 10 TA-AAbs, with the 5 remaining TA-AAbs made redundant due to overlap in ability to distinguish HCC from NCCLD controls. This panel of 10 TA-AAbs significantly improved the ability to distinguish HCC from NCCLD controls over any single TA-AAb alone with a sensitivity of 36.73% at a specificity of 89.90%. The Confirmation study panel of 10 TA-AAbs, identified by NRI, could distinguish HCC patients of all stages with no significant difference in sensitivity between early and late stage tumors. In comparison, the optimal panel in this study differs from the panel selected in our previous study [15]. This is primarily due to differences in sample cohort as well as overlap between patients positive for multiple TA-AAbs. Panel formation in relatively small patient cohorts, such as these, can lead to exclusion of biomarkers which show complete overlap with others in small patient cohorts but may not in a larger cohort. Results from this study and our previous study should be considered when designing a larger study with independent training and validation cohorts.

The 10 capture antigens for the TA-AAbs comprising the Confirmation study panel span multiple pathways and are primarily overexpressed in HCC. MAGE-A4, NY-ESO-1 and CAGE are cancer/testis antigens whose expression is normally restricted to germ cells [44], and have been found to be commonly ectopically expressed in HCC as well as other malignancies [45–48]. Overexpression of SOX2, EpCAM, RalA and SALL4B are associated with stem-cell characteristics and epithelial-mesenchymal transition (EMT), this overexpression is seen for many HCC patients [49–52]. Whilst not a marker of stemness, MMP9 is linked with EMT as it plays a key role in degradation of the extracellular matrix and therefore by aiding the escape of epithelial tumor cells from their location of origin, MMP9 expression has been linked to the metastatic potential of HCC cells [53]. AIF-1 has been shown to promote proliferation and migration of breast cancer cells [54,55], and whilst its role in HCC remains unknown, increased mRNA levels in HCC tumor cells have been observed [56]. Presence of TA-AAbs to the majority of these antigens in HCC patients have previously been described [15,20,57–59]. However, this is the first study to report the detection of autoantibodies to MMP9, AIF-1 and EpCAM in HCC patients.

We observe that TA-AAb levels are similar for the individual groups baring a few outliers (1–12%). This is a commonly observed feature of TA-AAb measurement within the literature [25,60,61]. This is primarily due to the amplified nature of an antibody response and the rarity of individual TA-AAbs to each TAA. As the antibody response is amplified rather than linked to the size/activity of a tumor, population means will be similar for each TAA with the few outliers representing the true amplified response. Testing at a single serum dilution as performed in this study potentially introduces limitations to the dynamic range of the assay. One serum dilution was used in this study to simplify the procedure allowing interrogation of a large number of markers. The dilution used has previously been shown to give reproducible results within a linear signal range in a TA-AAb based test for lung cancer [30]. Antigens were coated at multiple concentrations (50 and 160 nM) to enable selection of the optimal concentration for this serum dilution.

The improved performance for detection of HCC of TA-AAbs in combination with AFP compared with AFP alone has been demonstrated previously [28]. AFP performance in this study was within the ranges reported in the literature [62]. It is commonly reported for AFP to have low sensitivity in early stage HCC as AFP is only secreted by certain molecular subtypes of HCC [63], and in those which do, AFP levels positively correlate with tumor size, number, differentiation and vascular invasion [7]. Sensitivity of AFP was low when compared to the TA-AAb panel for stage one HCCs (17% vs 29%) but improved for later stages. Combination of AFP and the TA-AAb panel significantly increased the ability to distinguish HCC from NCCLD controls compared to the TA-AAb panel or AFP alone. Sensitivity of the TA-AAb panel plus AFP was significantly associated with increasing stage, however, the combination still outperformed either the TA-AAb panel or AFP alone for detection of stage 1 and 2 HCC.

The object of this study was primarily discovery, to assess an additional and different set of markers to our previous study [15]. This study therefore expands the knowledge base with which to develop a panel of markers which, due to the limited size of the cohorts used to date, will need to be verified by appropriately powered training and validation studies using independent patient cohorts in future studies. The Confirmation assay allowed us to test a combination of the best markers from this Discovery study alongside lead markers from our previous study [15] using independent batches of all the antigens. Despite the limited sample set, K-fold cross-validation produced a similar AUC as the whole sample set, 0.629 and 0.616 respectively, with a small standard deviation of 0.062 indicating a low level of data overfitting.

The NCCLD samples in this study were obtained as part of a routine check for the status of liver disease during a liver clinic with no follow-up data, therefore we do not know the number of control samples that may have been false positives with undetected HCC at the time of sampling. This could relate to an improvement in specificity in a clinical scenario, for example, if the specificity is recalculated to account for a 2.40% prevalence of HCC in the NCCLD cohort [38], specificity would rise to 90.54% for the TA-AAb panel and 91.00% for the TA-AAb plus AFP panel. This study is also restricted by the use of HCC samples from a commercial biobank with limited metadata which has prevented analysis of other clinically relevant factors such as Child-Pugh score and BCLC staging. However, we believe the sample collection for this study was an appropriate approach to gather data to inform the design of a study with prospective collection including extensive metadata.

The semi-systematic review of the literature used for biomarker identification in this study appears fit for purpose to expand on the data collected previously [15]. Future discovery studies could also encompass review of increasingly available next generation sequencing (NGS) data to determine likely TAAs by detecting tumor specific mutations and aberrantly expressed proteins which could elicit an immune response.

A cut-off of 200 ng/ml was chosen for AFP in this study. The HCC samples in this study were obtained commercially with limited diagnosis data. Whilst AFP is not recommended as a screening tool for HCC, it is often utilised in the monitoring of patients with liver disease. Therefore, if AFP was measured during the monitoring of the patients, this could have led to a bias towards patients with raised AFP, especially those with moderately raised AFP. A cut-off of 200 ng/ml is associated with high specificity for HCC which should alleviate bias, as samples with AFP greater than 200 ng/ml would likely have been diagnosed by traditional methods therefore, this cut-off is more likely to be reproducible in a real-world scenario. There is debate as to whether AFP is additive to US [64], however the differing biological pathways which trigger TA-AAb production and the increased sensitivity for early stage HCC, may lead to the TA-AAb plus AFP test being more complemental to US than AFP alone. HCC is very common in non-western countries where healthcare budgets are limited and access to health care clinics is often restricted. Sensitivity of the TA-AAb panel plus AFP is significantly better than that of AFP alone, as well as improving the sensitivity of detection of the earlier stages of disease which have the best chance of cure but which are most difficult to detect by traditional screening strategies. The potential stage shift plus the low sample volume requirements and low cost of the test, increase the likelihood of favourable cost-effectiveness. An alternative strategy could be implemented with the TA-AAb panel used only in AFP negative patients, especially if AFP is measured with a high specificity cut-off such as 200 ng/ml as used in this study. The TA-AAb panel had a sensitivity of 34.33% in AFP negative patients, and 27.59% in early stage AFP negative patients. This would therefore reduce the number of TA-AAb tests needed as any AFP positive patients would not receive the TA-AAb test but will still allow for detection of 27.59% of early stage tumors which would normally go undetected by AFP resulting in an addition of 18.82% of early stage tumors detected over AFP alone.

This study has established a panel of TA-AAbs which in combination with AFP could have clinical utility for aiding surveillance strategies or diagnosis of HCC. The early stage sensitivity of the combination test (45.45%) was similar to that of ultrasound reported in the literature (45%) [5], and can offer other advantages over US. One problem with US is operator variance dependent on experience and equipment available, as well as variance associated with patient factors such as underlying liver disease, cirrhosis status, gender and BMI [65]. Measurement of TA-AAbs has previously been shown to exhibit low variance in results [66] and interpretation is non-subjective as long as cut-off derivation is clearly explained and standardised. US is considered inexpensive when compared to other imaging modalities such as CT and MRI, however, as the detection of TA-AAbs in this study is based on a simple ELISA based test employing capture antigens produced in *E*. *coli* it offers an even cheaper alternative and indeed health economic studies have demonstrated cost effectiveness of a TA-AAb test in lung cancer patients [67]. This test as it stands does not outperform US so is unlikely to replace it completely, however follow-up studies should be undertaken to analyse the combination of the two tests in order to assess potential improvement in sensitivity.

This study has enabled identification of a TA-AAb panel ELISA which can distinguish HCC patients from NCCLD and healthy patients and may be additive to AFP for the detection of early stage disease. This work lays the foundations for further technical and clinical validation in independent cohorts to avoid bias and demonstrate clinical utility.

## Supporting information

S1 Table Search terms for antigen identification. (DOCX)

S2 Table Aetiology of the NCCLD controls. (DOCX)

S3 Table Performance of each of the 44 potential TA-AAbs identified in the literature search when tested on the Discovery cohort. Dashes indicate that no cut-off could be assigned under the given criteria. ^N^ and ^C^ indicate N-terminal and C-terminal tagged versions of the same antigen respectively.
(DOCX)

S4 Table Performance of each of the 21 potential TA-AAbs tested on the Confirmation cohort. Dashes indicate that no cut-off could be assigned under the given criteria.
(DOCX)
