# Supplementary material for: Tumor-associated autoantibodies in combination with alpha-fetoprotein for detection of early stage hepatocellular carcinoma
Source: PLoS One. 2020 May 6;15(5):e0232247. doi: 10.1371/journal.pone.0232247 (PMC7202612; doi:10.1371/journal.pone.0232247)
Supplement: S1 Table — (DOCX) [file pone.0232247.s001.docx]

**S1 Table. Search terms for Antigen identification.**

| **(i) Search for antigens with existing TA-AAb data in HCC** | (((Autoantibod*[Title/Abstract]) AND Hepatocellular carcinoma[Title/Abstract]) AND English[Language]) |
| --- | --- |
| **(ii) Search for review articles of proteins highly associated with HCC** | (((marker[Title/Abstract] OR biomarker[Title/Abstract])) AND (HCC[Title/Abstract] OR "primary liver cancer"[Title/Abstract])) AND (screening[Title/Abstract] OR diagnos*[Title/Abstract] OR prognos*[Title/Abstract]) published within the last 10 years |
