# Supplementary material for: Tumor-associated autoantibodies in combination with alpha-fetoprotein for detection of early stage hepatocellular carcinoma
Source: PLoS One. 2020 May 6;15(5):e0232247. doi: 10.1371/journal.pone.0232247 (PMC7202612; doi:10.1371/journal.pone.0232247)
Supplement: S2 Table — (DOCX) [file pone.0232247.s002.docx]

**S2 Table. Aetiology of the NCCLD controls.**

| **NCCLD controls** | **Discovery** | **Confirmation** |
| --- | --- | --- |
| Hepatitis C viral infection | 67 | 58 |
| Hepatitis B viral infection | 22 | 31 |
| Alcoholic Liver Disease | 6 | 6 |
| Autoimmune Hepatitis | 2 | 2 |
| Haemochromatosis | 1 | 1 |
| Primary Biliary Cirrhosis | 1 | 1 |
