# Supplementary material for: Tumor-associated autoantibodies in combination with alpha-fetoprotein for detection of early stage hepatocellular carcinoma
Source: PLoS One. 2020 May 6;15(5):e0232247. doi: 10.1371/journal.pone.0232247 (PMC7202612; doi:10.1371/journal.pone.0232247)
Supplement: S3 Table — Dashes indicate that no cut-off could be assigned under the given criteria. N and C indicate N-terminal and C-terminal tagged versions of the same antigen respectively. (DOCX) [file pone.0232247.s003.docx]

**S3 Table.** **Performance of each of the 44 potential TA-AAbs identified in the literature search when tested on the discovery cohort.**

| **Antigen** | **Concentration (nM)** | **Sensitivity (%)** | **Specificity NCCLD (%)** | **Specificity Healthy (%)** | **Youden’s J statistic** |
| --- | --- | --- | --- | --- | --- |
| AIF-1 | 160 | 2.02 | 97.98 | 98.99 | 0.00 |
| AIF-1 | 50 | 2.02 | 100.00 | 100.00 | 0.02 |
| AKR1B10 | 160 | 1.01 | 98.99 | 100.00 | 0.00 |
| AKR1B10 | 50 | 1.01 | 100.00 | 100.00 | 0.01 |
| AnnexinII | 160 | 1.01 | 98.99 | 100.00 | 0.00 |
| AnnexinII | 50 | 1.01 | 98.99 | 100.00 | 0.00 |
| APOA1 | 160 | 1.01 | 98.99 | 100.00 | 0.00 |
| APOA1 | 50 | 1.01 | 100.00 | 100.00 | 0.01 |
| BCL2 | 160 | 3.03 | 98.99 | 98.99 | 0.02 |
| BCL2 | 50 | 1.01 | 98.99 | 100.00 | 0.00 |
| CD44 | 160 | 1.01 | 100.00 | 100.00 | 0.01 |
| CD44 | 50 | 1.01 | 100.00 | 100.00 | 0.01 |
| CDKN1B | 160 | 7.07 | 95.96 | 93.94 | 0.03 |
| CDKN1B | 50 | 2.02 | 100.00 | 98.99 | 0.02 |
| CHGA | 160 | 1.01 | 98.99 | 98.99 | 0.00 |
| CHGA | 50 | 1.01 | 98.99 | 98.99 | 0.00 |
| CK18 | 160 | 1.01 | 98.99 | 100.00 | 0.00 |
| CK18 | 50 | 1.01 | 100.00 | 100.00 | 0.01 |
| CK19 | 160 | - | - | - | - |
| CK19 | 50 | - | - | - | - |
| CPS1-C | 160 | 2.02 | 98.99 | 100.00 | 0.01 |
| CPS1-C | 50 | 1.01 | 98.99 | 100.00 | 0.00 |
| CPS1-N | 160 | 1.01 | 98.99 | 100.00 | 0.00 |
| CPS1-N | 50 | 1.01 | 98.99 | 100.00 | 0.00 |
| DDX3X^C^ | 160 | 1.01 | 98.99 | 100.00 | 0.00 |
| DDX3X^C^ | 50 | 1.01 | 98.99 | 100.00 | 0.00 |
| DDX3X^N^ | 160 | 3.03 | 98.99 | 97.98 | 0.02 |
| DDX3X^N^ | 50 | 4.04 | 95.96 | 96.97 | 0.00 |
| EEF2 | 160 | 1.01 | 98.99 | 100.00 | 0.00 |
| EEF2 | 50 | 1.01 | 98.99 | 100.00 | 0.00 |
| EPCAM | 160 | 2.02 | 100.00 | 98.99 | 0.02 |
| EPCAM | 50 | 5.05 | 98.99 | 93.94 | 0.04 |
| F2 | 160 | - | - | - | - |
| F2 | 50 | 1.01 | 98.99 | 100.00 | 0.00 |
| FUCA1 | 160 | - | - | - | - |
| FUCA1 | 50 | 1.01 | 100.00 | 100.00 | 0.01 |
| GLUL | 160 | - | - | - | - |
| GLUL | 50 | 1.01 | 100.00 | 100.00 | 0.01 |
| GOLM1 | 160 | 2.02 | 97.98 | 97.98 | 0.00 |
| GOLM1 | 50 | 1.01 | 98.99 | 98.99 | 0.00 |
| HNRNPA2 | 160 | 1.01 | 100.00 | 100.00 | 0.01 |
| HNRNPA2 | 50 | 1.01 | 100.00 | 100.00 | 0.01 |
| HNRNPL | 160 | 3.03 | 97.98 | 100.00 | 0.01 |
| HNRNPL | 50 | - | - | - | - |
| HSPA2 | 160 | 1.01 | 100.00 | 100.00 | 0.01 |
| HSPA2 | 50 | - | - | - | - |
| HSPA4 | 160 | 5.05 | 98.99 | 100.00 | 0.04 |
| HSPA4 | 50 | 4.04 | 100.00 | 100.00 | 0.04 |
| HSPD1 | 160 | 2.02 | 97.98 | 100.00 | 0.00 |
| HSPD1 | 50 | 2.02 | 97.98 | 100.00 | 0.00 |
| IL-6 | 160 | 2.02 | 97.98 | 98.99 | 0.00 |
| IL-6 | 50 | 2.02 | 97.98 | 97.98 | 0.00 |
| IL-8 | 160 | 4.04 | 97.98 | 94.95 | 0.02 |
| IL-8 | 50 | 5.05 | 95.96 | 95.96 | 0.01 |
| KRT23 | 160 | 1.01 | 98.99 | 97.98 | 0.00 |
| KRT23 | 50 | 1.01 | 98.99 | 100.00 | 0.00 |
| LMNB1 | 160 | 2.02 | 97.98 | 97.98 | 0.00 |
| LMNB1 | 50 | 1.01 | 98.99 | 100.00 | 0.00 |
| MDM2 | 160 | 2.02 | 98.99 | 98.99 | 0.01 |
| MDM2 | 50 | 2.02 | 98.99 | 100.00 | 0.01 |
| MMP9 | 160 | 3.03 | 98.99 | 98.99 | 0.02 |
| MMP9 | 50 | 1.01 | 98.99 | 100.00 | 0.00 |
| NPM1 | 160 | 2.02 | 98.99 | 100.00 | 0.01 |
| NPM1 | 50 | 1.01 | 100.00 | 100.00 | 0.01 |
| NY-ESO-1 | 160 | 7.07 | 100.00 | 98.99 | 0.07 |
| NY-ESO-1 | 50 | 8.08 | 98.99 | 96.97 | 0.07 |
| PEBP1 | 160 | 1.01 | 100.00 | 100.00 | 0.01 |
| PEBP1 | 50 | 1.01 | 100.00 | 100.00 | 0.01 |
| PRL | 160 | 2.02 | 98.99 | 98.99 | 0.01 |
| PRL | 50 | 1.01 | 100.00 | 100.00 | 0.01 |
| RGN | 160 | 1.01 | 100.00 | 100.00 | 0.01 |
| RGN | 50 | 1.01 | 100.00 | 100.00 | 0.01 |
| SALL4B | 160 | 2.02 | 97.98 | 100.00 | 0.00 |
| SALL4B | 50 | 1.01 | 100.00 | 100.00 | 0.01 |
| SPP1 | 160 | 1.01 | 98.99 | 98.99 | 0.00 |
| SPP1 | 50 | 1.01 | 98.99 | 100.00 | 0.00 |
| SSX2 | 160 | 3.03 | 97.98 | 96.97 | 0.01 |
| SSX2 | 50 | 3.03 | 98.99 | 98.99 | 0.02 |
| TF | 160 | 2.02 | 100.00 | 100.00 | 0.02 |
| TF | 50 | 2.02 | 98.99 | 98.99 | 0.01 |
| TGFB1 | 160 | 2.02 | 97.98 | 98.99 | 0.00 |
| TGFB1 | 50 | 1.01 | 100.00 | 100.00 | 0.01 |
| VIM | 160 | 7.07 | 96.97 | 93.94 | 0.04 |
| VIM | 50 | 3.03 | 98.99 | 95.96 | 0.02 |
| XRCC5 | 160 | - | - | - | - |
| XRCC5 | 50 | 1.01 | 98.99 | 100.00 | 0.00 |
| YWHAZ | 160 | 2.02 | 98.99 | 97.98 | 0.01 |
| YWHAZ | 50 | 1.01 | 98.99 | 100.00 | 0.00 |

Dashes indicate that no cut-off could be assigned under the given criteria. ^N^ and ^C^ indicate N-terminal and C-terminal tagged versions of the same antigen respectively.
