# Supplementary material for: Tumor-associated autoantibodies in combination with alpha-fetoprotein for detection of early stage hepatocellular carcinoma
Source: PLoS One. 2020 May 6;15(5):e0232247. doi: 10.1371/journal.pone.0232247 (PMC7202612; doi:10.1371/journal.pone.0232247)
Supplement: S4 Table — Dashes indicate that no cut-off could be assigned under the given criteria. (DOCX) [file pone.0232247.s004.docx]

**S4 Table. Performance of each of the 21 potential TA-AAbs tested on the confirmation cohort**.

| **Antigen** | **Concentration (nM)** | **Sensitivity (%)** | **Sensitivity AFP negative (%)** | **Specificity NCCLD (%)** | **Specificity Healthy (%)** | **Youden’s J statistic** |
| --- | --- | --- | --- | --- | --- | --- |
| AIF-1 | 160 | 1.02 | 0.00 | 100.00 | 100.00 | 0.01 |
| AIF-1 | 50 | 1.02 | 0.00 | 100.00 | 100.00 | 0.01 |
| CAGE | 160 | 9.18 | 7.46 | 97.98 | 93.68 | 0.07 |
| CAGE | 50 | 12.24 | 11.94 | 96.97 | 90.53 | 0.09 |
| CDKN1B | 160 | - | - | - | - | - |
| CDKN1B | 50 | - | - | - | - | - |
| DDX3X^N^ | 160 | 5.10 | 2.99 | 97.98 | 97.89 | 0.03 |
| DDX3X^N^ | 50 | 3.06 | 1.49 | 97.98 | 98.95 | 0.01 |
| EPCAM | 160 | 4.08 | 4.48 | 97.98 | 94.74 | 0.02 |
| EPCAM | 50 | 3.06 | 4.48 | 100 | 96.84 | 0.03 |
| GBU4-5 | 160 | - | - | - | - | - |
| GBU4-5 | 50 | - | - | - | - | - |
| HNRNPA2 | 160 | 6.12 | 5.97 | 95.96 | 93.68 | 0.02 |
| HNRNPA2 | 50 | - | - | - | - | - |
| HNRNPL | 160 | - | - | - | - | - |
| HNRNPL | 50 | - | - | - | - | - |
| HSPA4 | 160 | 4.08 | 1.59 | 98.99 | 100.00 | 0.03 |
| HSPA4 | 50 | 4.08 | 1.49 | 98.99 | 100.00 | 0.03 |
| HSPD1 | 160 | - | - | - | - | - |
| HSPD1 | 50 | - | - | - | - | - |
| MAGEA4 | 160 | 3.06 | 2.99 | 100.00 | 100.00 | 0.03 |
| MAGEA4 | 50 | 4.08 | 2.99 | 98.99 | 100.00 | 0.03 |
| MMP9 | 160 | 2.04 | 1.49 | 98.99 | 100.00 | 0.01 |
| MMP9 | 50 | 5.10 | 5.97 | 95.96 | 94.74 | 0.01 |
| NPM1 | 160 | - | - | - | - | - |
| NPM1 | 50 | - | - | - | - | - |
| NY-ESO-1 | 160 | 12.24 | 7.46 | 95.96 | 85.26 | 0.08 |
| NY-ESO-1 | 50 | 8.16 | 4.48 | 100.00 | 95.79 | 0.08 |
| P62 | 160 | - | - | - | - | - |
| P62 | 50 | 2.04 | 0.00 | 100.00 | 100.00 | 0.02 |
| RALA | 160 | 3.06 | 2.99 | 98.99 | 97.89 | 0.02 |
| RALA | 50 | 4.08 | 4.48 | 98.99 | 95.79 | 0.03 |
| SALL4B | 160 | 6.12 | 7.46 | 96.97 | 98.95 | 0.03 |
| SALL4B | 50 | 5.1 | 5.97 | 96.97 | 97.89 | 0.02 |
| SOX2 | 160 | 3.06 | 2.99 | 100.00 | 98.95 | 0.03 |
| SOX2 | 50 | 5.1 | 5.97 | 98.99 | 95.79 | 0.04 |
| TF | 160 | - | - | - | - | - |
| TF | 50 | 4.08 | 4.48 | 96.97 | 98.95 | 0.01 |
| VIM | 160 | - | - | - | - | - |
| VIM | 50 | - | - | - | - | - |
| YWHAZ | 160 | 6.12 | 5.97 | 95.96 | 94.74 | 0.02 |
| YWHAZ | 50 | - | - | - | - | - |

Dashes indicate that no cut-off could be assigned under the given criteria. Sensitivity in AFP negative indicates the sensitivity for HCC in patients with an AFP<200 ng/ml.
